# Exergetic Performance Coefficient Analysis and Optimization of a High-Temperature Proton Exchange Membrane Fuel Cell

**DOI:** 10.3390/membranes12010070

**Published:** 2022-01-05

**Authors:** Dongxu Li, Yanju Li, Zheshu Ma, Meng Zheng, Zhanghao Lu

**Affiliations:** College of Automobile and Traffic Engineering, Nanjing Forestry University, Nanjing 210037, China; lidongxu@njfu.edu.cn (D.L.); liyanju@njfu.edu.cn (Y.L.); zhengmeng@njfu.edu.cn (M.Z.); luzhanghao@njfu.edu.cn (Z.L.)

**Keywords:** HT-PEMFC, exergetic performance coefficient, performance optimization

## Abstract

Performance of a high-temperature proton exchange membrane fuel cell (HT-PEMFC) and the influence of different parameters on HT-PEMFC is analyzed in this study. Firstly, mathematical expression for energy efficiency, power density, exergy destruction and exergetic performance coefficient (*EPC*) are derived. Then, the relationship between the dimensionless power density, exergy destruction rate, exergetic performance coefficient (*EPC*) and energy efficiency is compared. Furthermore, the effect of flow rate, doping level, inlet pressure and film thickness are considered to evaluate the performance of HT-PEMFC. Results show that *EPC* not only considers exergetic loss rate to minimize exergetic loss, but also considers the power density of HT-PEMFC to maximize its power density and improve its efficiency, so *EPC* represents a better performance criterion. In addition, increasing inlet pressure and doping level can improve *EPC* and energy efficiency, respectively.

## 1. Introduction

Among fuel cell types, the proton exchange membrane fuel cell (PEMFC) has been widely used in mobile units and automobiles. A low-temperature proton exchange membrane fuel cell (LT-PEMFC) operates at around 80 °C and is used in fuel cell vehicles. Compared with LT-PEMFC, an HT-PEMFC operates at 120–200 °C and has a lot of advantages, such as higher CO tolerance [1,2], simplified water and heat management [3] and enhanced kinetics [4]. Hence, many studies [5,6,7,8,9,10] have been devoted to the HT-PEMFC.

Finite time thermodynamics (FTT) has been used to analyze various thermodynamic processes and cycles [11,12,13,14,15,16,17,18,19,20,21] for several decades. The electrochemical model of PEMFC can also be embodied into corresponding FTT model for thermodynamic performance analysis and optimization to pursue maximum performances under operation conditions. Currently, typical optimal FTT objective functions include exergy loss [22], exergy efficiency [23,24,25,26], ecological performance coefficient [14,20,26,27,28,29,30,31,32,33,34,35,36], ecological function [14] and entropy production rate [22,26]. Watowich et al. [37] used the optimal control theory to determine the limit of the fuel cell operation process. Sieniutycz et al. [38,39] established a steady-state model of the fuel cell and predicted the maximum power output from the perspective of thermodynamic optimization. Liu et al. [25] developed a PEMFC power system and established a FTT model including exergy destruction, exergy efficiency and ecological function et. al. Results showed that fuel cell stack and heat exchanger were the two components that caused the greatest exergy loss. Low current density can improve the ecological performance and the power and exergetic efficiency of the system, but reduce the exergy loss and the net power of the system. Ye et al. [23] analyzed the performance of an HT-PEMFC under different operating conditions by exergy analysis, and the results showed that higher operating temperature was beneficial to improve the efficiency and power of the system, but relative humidity and operating pressure had little influence on the system. Li et al. [14] analyzed the ecological performance of PEMFC and obtained the ecological performance coefficient ECOP=P/Tσ and ecological objective function ECOP=P−Tσ, which were defined as the ratio of power to power loss and the difference between power and power loss respectively. Guo et al. [24] obtained the optimal operation region of an HT-PEMFC under different parameters based on the maximum power density and did not compare different performance indexes and determine the optimal performance indexes. Akkaya et al. [40] defined a novel exergetic performance coefficient (*EPC)* which was used to analyze the performances of solid oxide fuel cell (SOFC) and compare it with other performance criteria. It is a thermal-ecological index that combines the functions of energy and exergy parameters. Therefore, *EPC* can be expected to better evaluate thermodynamic processes and cycles including HT-PEMFCs than the ecological performance coefficient and ecological objective function.

The purpose of this study is to evaluate HT-PEMFC by exergetic performance coefficient (*EPC*) and compare it with other FTT indexes. Firstly, the HT-PEMFC model is established which takes three kinds of polarization losses and leakage current density into account. Then, the relationships between *EPC*, power density, exergy destruction rate and energy efficiency are obtained. Finally, inlet pressure, doping level and film thickness are taken as important operating parameters to evaluate their influence on HT-PEMFC performances.

## 2. Exergetic Performance Coefficient (*EPC*) of High-Temperature Proton Exchange Membrane Fuel Cell (HT-PEMFC)

### 2.1. Working Principle of HT-PEMFC

**Figure 1:**
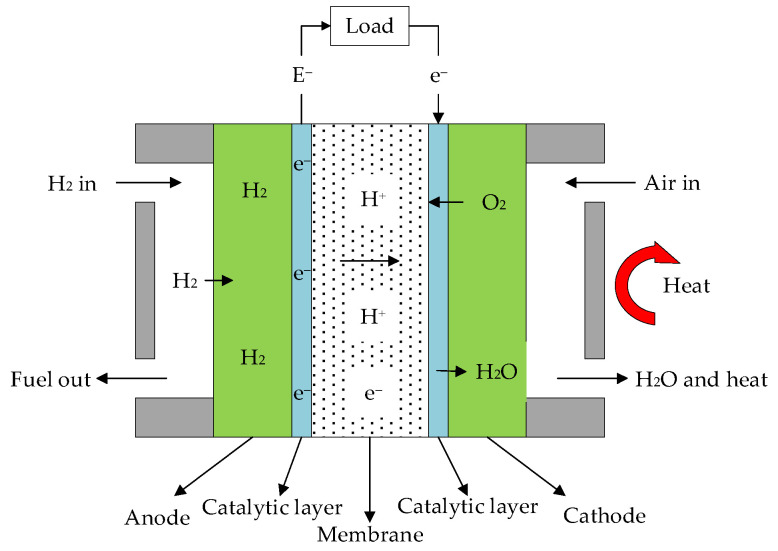
Working principle of high-temperature proton exchange membrane fuel cell (HT-PEMFC).

The structure and working principle of HT-PEMFC are shown in Figure 1 [26], which mainly includes an anode, cathode, electrolyte and external load. The electrochemical reaction equations of the HT-PEMFC are:

(1) $$\mathrm{Anode}\;\mathrm{rection}:{\;H}_{2}\to 2H^{+}+2e^{-}$$

(2) $$\mathrm{Cathodic}\;\mathrm{reaction}:\;2H^{+}+\frac{1}{2}O_{2}+2e^{-}\to H_{2}O+\mathrm{heat}$$

(3) $$\mathrm{Total}\;\mathrm{reaction}:{\;H}_{2}+\frac{1}{2}O_{2}\to H_{2}O+\mathrm{heat}+\mathrm{electricity}$$

According to the working principle of HT-PEMFC, the hydrogen and air flow rate can be expressed as follows:

(4) $$\dot{m_{H_{2}}}=ST_{a}\frac{jA}{2F}$$

(5) $$\dot{m_{\mathrm{air}}}=ST_{c}\frac{jA}{4\cdot 0.21\cdot F}$$

where $ST_{a}$ and $ST_{c}$ are hydrogen stoichiometry and air stoichiometry respectively; j is current density; A is the cell active area; F is Faraday constant.

### 2.2. Thermodynamic Model of HT-PEMFC

The HT-PEMFC model is formulated on the basis of the following assumptions:

(1) The HT-PEMFC system is working under steady-state conditions;
(2) Kinetic and potential energy are neglected;
(3) All gases within the HT-PEMFC are assumed to be ideal gas;
(4) The environment condition is 1.013 bar (1 atm) and 25 °C; air consists of 79% nitrogen and 21% oxygen;
(5) Anode outlet temperature is equal to the operating temperature;
(6) There is no leakage of hydrogen and oxygen within the HT-PEMFC structure.

For the HT-PEMFC, reversible potential can be given as Equation (6):

(6) $$E_{r}=E_{r}^{0}+\frac{\Delta S}{nF}\left(T-T_{0}\right)+\frac{RT}{nF}\ln(\frac{p_{H_{2}}p_{O_{2}}^{0.5}}{p_{H_{2}O}})$$

In Equation (6), $E_{r}^{0}$ is the ideal standard potential [26]; ΔS is the change of standard molar entropy [26]; T is the operating temperature of HT-PEMFC; *R* is the gas constant.

For the HT-PEMFC, due to three types of overpotential which includes activation overpotential $E_{act}$, concentration overpotential $E_{con}$ and ohmic overpotential $E_{ohm}$, its actual output voltage is generally less than the reversible [26]. The actual output voltage can be shown as Equation (7):

(7) $$E_{cell}=E_{r}-E_{act}-E_{ohm}-E_{con}$$

The power density of the HT-PEMFC can be derived as:

(8) $$P=E_{cell}\cdot j\cdot A$$

where j is current density and A is the electrode effective surface area.

As an energy conversion device, the output efficiency [26] of HT-PEMFC can be shown as Equation (9):

(9) $$\eta =-\frac{P}{\Delta \dot{H}}$$

where $\Delta \dot{H}$ is the total energy absorbed from hydrogen and oxygen [26], it can be expressed as:

(10) $$\Delta \dot{H}=-\frac{jA\Delta h}{nF}$$

where Δh is the change of molar enthalpy.

The heat leakage rate [24] from HT-PEMFC to the environment can be represented by:

(11) $${\dot{Q}}_{L}=K_{L}A_{L}\left(T-T_{0}\right)$$

where $K_{L}$ and $A_{L}$ represent the heat leakage coefficient and the corresponding heat leakage area, respectively. T and $T_{0}$ are, respectively, the temperature of the HT-PEMFC and the environment.

According to the first law of thermodynamics, the remaining part of the thermal rate from the HT-PEMFC can be expressed as:

(12) $${\dot{Q}}_{H}=-\Delta \dot{H}-P-{\dot{Q}}_{L}=\frac{A}{nF}\left[-\left(1-\eta \right)j\Delta h-b_{1}\left(T-T_{0}\right)\right]$$

where $b_{1}=\frac{nFK_{L}A_{L}}{A}$ [24,41].

### 2.3. Exergetic Performance Analysis

The output performance of HT-PEMFC is degraded due to different irreversibilities including heat loss, friction between gas and channel, fuel mixing, leakage current, electrochemical reaction, and polarization. Exergetic analysis provides a standard for evaluating the quality of energy released by HT-PEMFC and can represent the actual useful work of energy.

**Figure 2:**
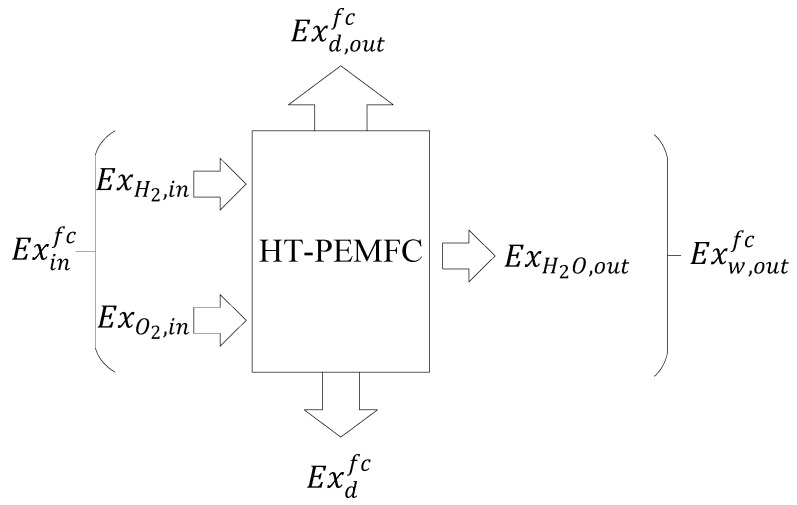
Exergetic equilibrium of the HT-PEMFC.

The exergetic equilibrium equation of HT-PEMFC includes the total exergy of hydrogen and oxygen $Ex_{in}^{fc}$, output power of HT-PEMFC $Ex_{d,out}^{fc}$, exergetic loss generated by water $Ex_{w,out}^{fc}$, and irreversible heat loss $Ex_{d}^{fc}$. Figure 2 shows the exergetic equilibrium of HT-PEMFC. The exergetic equilibrium equation of HT-PEMFC is shown as Equation (13) [40]:

(13) $$Ex_{in}^{fc}=Ex_{d,out}^{fc}+Ex_{w,out}^{fc}+Ex_{d}^{fc}$$

For HT-PEMFC, only physical exergy and chemical exergy is considered in the exergy of HT-PEMFC reaction process. The expression of the total exergy can be written as:

(14) $$ex={\left(ex\right)}^{ph}+{\left(ex\right)}^{ch}$$

Physical exergy [42,43] and chemical exergy [22,42] are expressed as:

(15) $${\left(ex\right)}^{ph}=\left(h-h_{0}\right)-T_{0}\left(s-s_{0}\right)$$

(16) $${\left(ex\right)}^{ch}=\sum x_{n}\cdot e_{n}^{ch}+RT_{0}\sum x_{n}\cdot lnx_{n}\;$$

where $x_{n}$ and $e_{n}^{ch}$ are the molar fraction and the standard chemical exergy of $H_{2}$, $O_{2}$ and $H_{2}O$, respectively.

In HT-PEMFC, the total input exergy rate and the output exergy rate are shown as follows:

(17) $${\dot{ex}}_{in}=\frac{jA}{nF}\left(ex_{H_{2}}+0.5ex_{O_{2}}\right)$$

(18) $${\dot{ex}}_{out}=\frac{jA}{nF}ex_{H_{2}O}$$

In practical application, energy depreciation is inevitable, including heat transfer, chemical reaction friction and polarization. These irreversibilities will lead to exergetic loss. Exergetic loss can be used to measure the degree of irreversibility of the process. The greater exergetic loss, the greater irreversibility of HT-PEMFC thermodynamic process, and the less effective utilization of exergy. Exergy destruction rate of the HT-PEMFC can be expressed as [43]:

(19) $$ExD={\dot{ex}}_{in}-{\dot{ex}}_{out}-P+{\dot{Q}}_{H}\left(1-T_{0}/T\right)$$

In the HT-PEMFC system, energy analysis can provide necessary fuel consumption information, while exergetic performance analysis can evaluate the impact of irreversibility in the system. From the perspective of better performance and ecology, an evaluation index including energy and exergetic performance is defined as an alternative standard, which is more conducive to engineering decisions and can be derived as [40]:

(20) $$EPC=\frac{P}{ExD}=\frac{E_{cell}\cdot j\cdot A}{{\dot{ex}}_{in}-{\dot{ex}}_{out}-P+{\dot{Q}}_{H}\left(1-T_{0}/T\right)}$$

At the same time, due to the numerical differences among various indicators, in order to better compare the relationship between EPC, P, η and ExD, dimensionless method is adopted for the numerical calculation and analysis. The dimensionless function of EPC, P, ExD and η are $\bar{EPC}=EPC/EPC_{max}$, $\bar{P}=P/P_{max}$, $\bar{ExD}=ExD/ExD_{max}$ and $\bar{\;\eta }=\eta /\eta_{max}$.

### 2.4. Optiaml Exergy Performance Coefficient

In order to find the performance limit of HT-PEMFC and the balance between energy and exergy, this paper optimized the dimensionless power density, efficiency, exergy loss rate and *EPC* of HT-PEMFC [44,45,46,47,48,49]. The doping level (DL), relative humidity (RH), inlet pressure (p) and film thickness $\left(t_{mem}\right)$ corresponding to the optimal performance of HT-PEMFC are obtained.

The current density, inlet pressure, film thickness and doping level are taken as the constraints condition of the dimensionless EPC. The expression is $\bar{EPC}=f\left(j,\;p,\;RH,DL,t_{mem}\right)$. When $j,\;p,\;RH,\;t_{mem}$ are constant, the expression is $\bar{EPC}=g\left(DL\right)$.

Therefore, the value of $DL_{opt}$ can be determined under optimal *EPC* performance. However, using mathematical method and numerical algorithm, selecting EPC as objective function when $j,DL,\;RH,t_{mem}$ or j,DL, RH,p is given, with the constraints p or $t_{mem}$, one can obtain the optimal *EPC* at an optimal $p_{opt}$ or $t_{mem_{opt}}$.

## 3. Results and Discussion

The relevant parameters used in the HT-PEMFC model refer to the literature [22,26] as shown in Table 1. Moreover, parametric studies are performed to investigate the HT-PEMFC performance. According to Refs. [21,22,26], the variation ranges of operating pressure p, doping level DL and film thickness $t_{mem}$ are listed in Table 2.

### 3.1. Comparsion of Relationship between EPC,P,ExD and η

**Figure 3:**
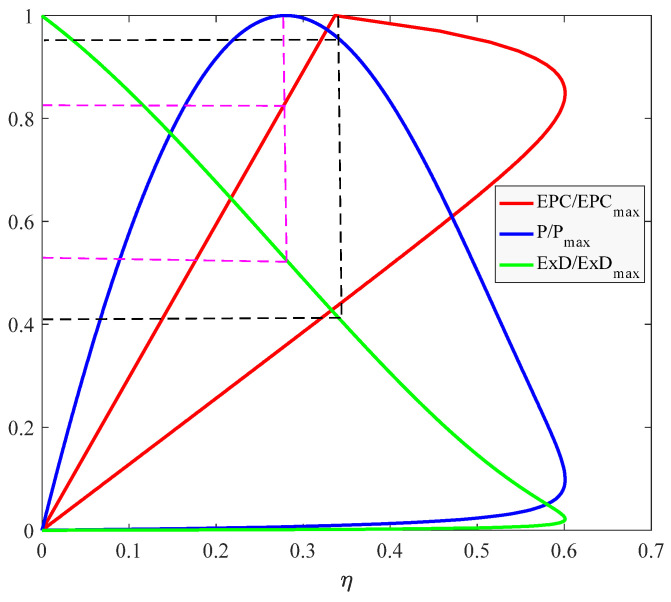
Relationship between efficiency and dimensionless EPC, P, ExD.

It can be seen from Figure 3 when EPC reaches the maximum value, the efficiency is 0.34, $ExD/ExD_{max}$ is 0.4 and $P/P_{max}$ is 0.9414. This means the power density at $EPC_{max}$ is very close to the maximum. When P is at its maximum value, the efficiency is 0.27, $ExD/ExD_{max}$ is 0.53 and $EPC/EPC_{max}$ is 0.83. Obviously, compared to $P_{max}$, if $EPC_{max}$ is taken as the criterion, exergetic loss rate decreases by 23%, efficiency increases by 23%, power density only decreases by 5%. Therefore, the EPC index is derived in this paper, which not only considers exergetic loss rate to minimize exergetic loss, but also considers the power density of HT-PEMFC to maximize its power and improve its efficiency.

### 3.2. Influence of Inlet Flow Rate on HT-PEMFC

**Figure 4:**
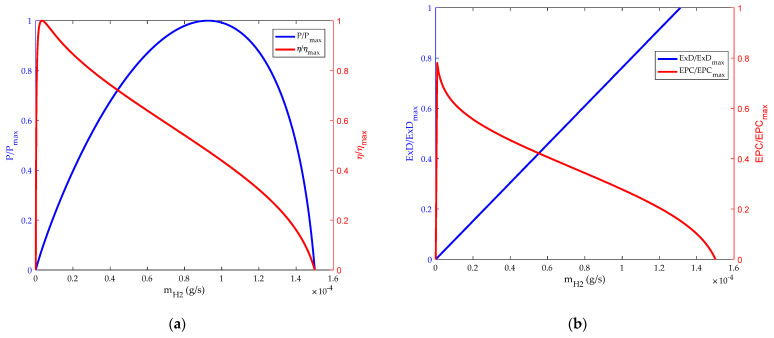
Influence of hydrogen flow rate. (**a**) Power density and energy efficiency; (**b**) exergy destruction rate and exergetic performance coefficient.

**Figure 5:**
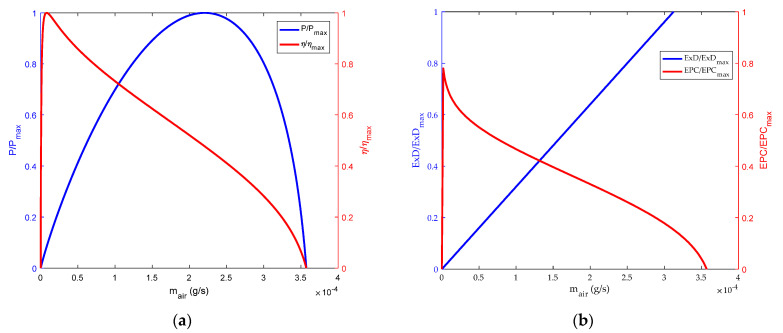
Influence of air flow rate. (**a**) power density and energy efficiency; (**b**) exergy destruction rate and exergetic performance coefficient.

The changes of energy and exergetic performance based on hydrogen flow and air flow are shown in Figure 4 and Figure 5. It can be seen from Figure 4a and Figure 5a that the thermodynamic efficiency of HT-PEMFC has a maximum value in the region of low hydrogen and air flow. This is because in the initial stage the electrochemical reaction rate accelerates, and the power loss caused by polarization is small. With the increase of hydrogen and air flow, the thermodynamic efficiency of the HT-PEMFC will decrease. However, the power density will reach its maximum value at a specific hydrogen and oxygen flow rate. The main reason is that the output power of the HT-PEMFC increases with the increase of hydrogen and air flow rate. When the hydrogen and air flow rate are too high, the polarization phenomenon of the fuel cell is obvious, so the output voltage and power density decrease. Figure 4b and Figure 5b show the influence of hydrogen and air flow on *EPC* and exergy destruction rate. It can be seen that the value of *EPC* will reach the maximum at the initial stage and then decrease gradually. As can be seen from the figure, the curve characteristics of *EPC* and thermodynamic efficiency have similar trends, but they carry different implications. Thermodynamic efficiency represents the amount of fuel consumed to produce a certain amount of power, while *EPC* represents the destruction rate of availabilities.

### 3.3. Influence of Doping Level on HT-PEMFC

**Figure 6:**
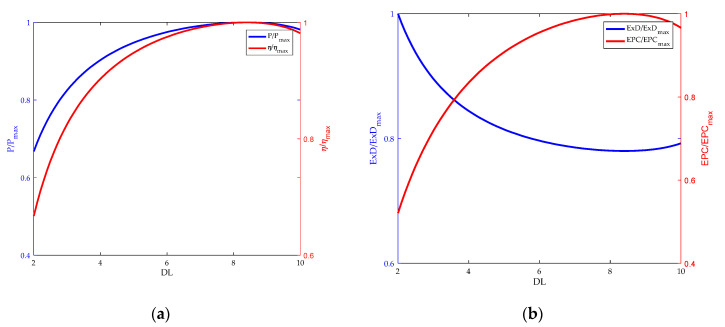
Influence of doping level. (**a**) Power density and energy efficiency; (**b**) exergy destruction rate and exergetic performance coefficient.

The HT-PEMFC using the polybenzimidazole membrane doped with phosphoric acid molecules as electrolyte are studied in this paper. The doping level (*DL*) is the number of phosphoric acid molecules per polybenzimidazole (PBI). *DL* plays an important role in the ohmic overpotential. The relationship between the performance of HT-PEMFC and the doping level (*DL*) is shown in Figure 6. As the *DL* increases, the power density and energy efficiency reach the maximum value. When *DL* is further increased, the power density and energy efficiency decrease. When *DL* is 8, both power density and efficiency reach maximum values. As can be seen from Figure 6b, *EPC* acts as energy efficiency. However, when the exergy destruction rate reaches the minimum value, *EPC* is the maximum value. When energy efficiency and *EPC* are both maximized, the doping level is the same value. This is an expected result. Since minimum fuel consumption yields the maximum power at a certain power, environmental pollution is minimal, consistent with the maximum *EPC*. Hence, the optimal doping level is 8 in terms of EPC, P, ExD and η.

### 3.4. Influence of Inlet Pressure on HT-PEMFC

**Figure 7:**
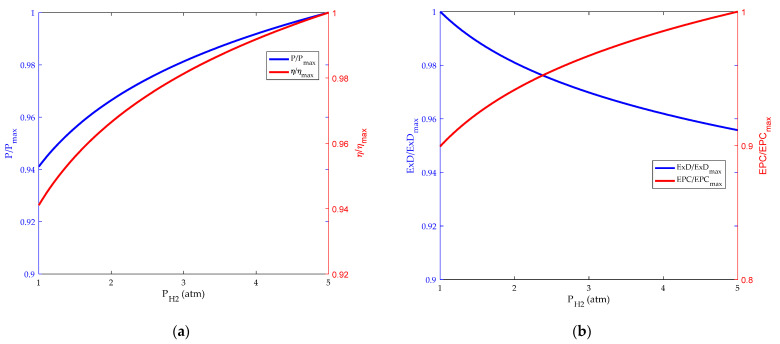
Influence of hydrogen pressure. (**a**) Power density and energy efficiency; (**b**) exergy destruction rate and exergetic performance coefficient.

**Figure 8:**
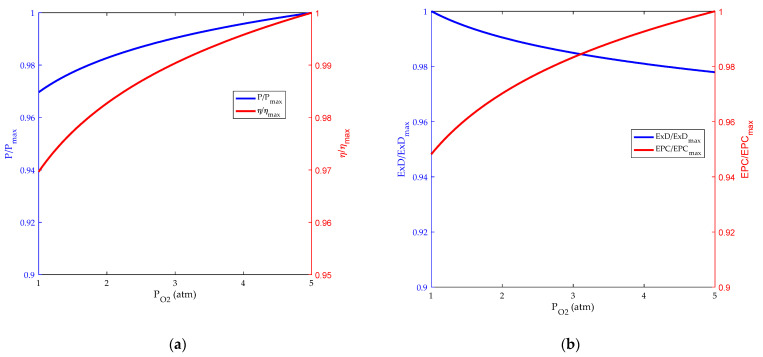
Influence of oxygen pressure. (**a**) Power density and energy efficiency; (**b**) exergy destruction rate and exergetic performance coefficient.

The inlet pressure is an important factor that affects the performance of HT-PEMFC. Figure 7 and Figure 8, respectively, represent the effects of hydrogen and oxygen inlet pressure on HT-PEMFC performance. As can be seen from the figure, both the power density and efficiency of HT-PEMFC increase as the hydrogen and oxygen inlet pressure increase from 1 to 5 atm with a step width of 1 atm [50], but the increase is not numerically significant. Increasing the inlet pressure, on the one hand, increases the diffusion rate of the gas, improves the mass transfer of the reaction gas. On the other hand, it increases the concentration of gas and reduces the influence of concentration polarization on reversible potential.

### 3.5. Influence of Film Thickness on HT-PEMFC

**Figure 9:**
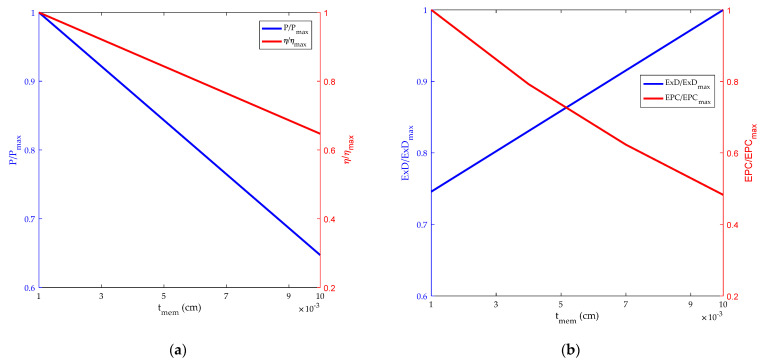
Influence of film thickness. (**a**) Power density and energy efficiency; (**b**) exergy destruction rate and exergetic performance coefficient.

Figure 9 reflects the impact of $t_{mem}$ on HT-PEMFC power, efficiency, exergy destruction rate and *EPC*. It is obvious that the power, efficiency and *EPC* all improve with the decrease of $t_{mem}$, mainly because of the thinning of the membrane and the path length of the ions between anode and cathode is reduced, resulting in the decrease of ohmic potential of HT-PEMFC. However, if the membrane is too thin, it will cause fuel penetration, short circuit, film rupture and other problems. Therefore, it is essential to select the high temperature membrane in a proper range.

### 3.6. Relationship between EPC and Efficiency

**Figure 10:**
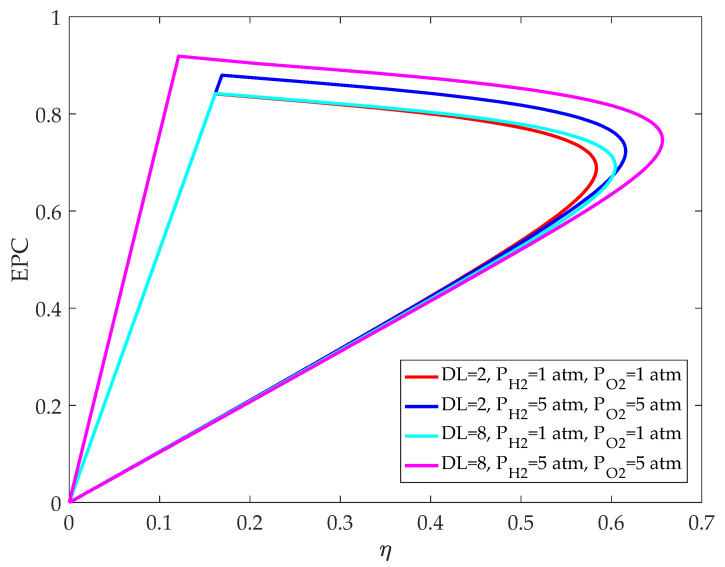
Exergetic performance coefficient versus energy efficiency before and after optimizing.

Figure 10 shows the relationship between *EPC* and efficiency before and after optimization of HT-PEMFC parameters. It can be seen that when *DL* and the efficiency are the same, increasing the inlet pressure can significantly improve the *EPC* of HT-PEMFC, indicating that the operating pressure has an impact on the power density and exergy destruction rate. When the operating pressure is the same, it is obvious that the efficiency of HT-PEMFC is improved by optimizing *DL*. The efficiency and *EPC* of HT-PEMFC are significantly improved by increasing both the operating pressure and *DL*, which not only reduces fuel consumption, but also reduces energy loss. However, if *DL* is too high, the mechanical property will become worse and the phosphoric acid molecules will leak out of the HT-PEM more easily. Besides, a higher inlet pressure consumes additional power to compress the inlet reactants.

In practical application, when HT-PEMFC is applied to vehicles, the *EPC* and energy efficiency of HT-PEMFC can be improved by increasing inlet pressure and doping level. When *EPC* increases, it shows that HT-PEMFC can produce more energy by causing less dissipation in the environment. Moreover, as the energy efficiency increases, it indicates that the HT-PEMFC consumes less fuel for the same amount of energy. Therefore, the HT-PEMFC performs better in terms of power output and the ecological environment, taking into account better *EPC* and efficiency operating conditions.

## 4. Conclusions

The performance of an HT-PEMFC is analyzed based on a new standard named exergetic performance coefficient, which is defined as the ratio of power to exergy destruction rate. The relationship between exergetic performance coefficient (*EPC)*, power density, exergy destruction rate and energy efficiency is obtained, and the result shows that *EPC* can replace power density as a new performance criterion. In the analysis of the HT-PEMFC model, the influence of different parameters on power density, energy efficiency, exergy destruction rate and *EPC* is analyzed. It is found that *EPC* and energy efficiency have the same trend, but different meanings. The $EPC_{max}$ represents the most energy obtained from the HT-PEMFC while causing the least dissipation in the environment. Therefore, the higher the *EPC* of the HT-PEMFC, the better the performance in terms of power and ecological environment. In addition, improving the inlet pressure can significantly boost *EPC*, and increasing *DL* can greatly enhance energy efficiency. For future research in the field of engineering, this new criterion can be used to analyze fuel cell vehicles.

## Figures and Tables

**Table 1:** Parameters used in the modeling.

Parameter	Value
Faraday constant, $F\;\left({C\;\mathrm{mol}}^{-1}\right)$	96,485
Gas constant, $R\;\left({J\;\mathrm{mol}}^{-1}{\;K}^{-1}\right)$	8.314
Number of electrons, n	2
Operating temperature, T (K)	438 [22]
Anode pressure *(*atm*)*	1 [22]
Cathode pressure *(*atm*)*	1 [22]
Anode gas compositions	100% $H_{2}$ [22]
Cathode gas compositions	21% $O_{2}/79\%\;N_{2}$ [22]
The doping level, DL	10 [26]
The relative humidity, RH	3.8% [26]
Thickness of the electrolyte, $t_{mem}\;\left(\mathrm{cm}\right)$	0.005 [26]

**Table 2:** Variation ranges of p, DL and $t_{mem}$.

Parameter	Value
Hydrogen pressure, $p_{H_{2}}\;\left(\mathrm{atm}\right)$	1–5 [21]
Oxygen pressure, $p_{O_{2}}\;\left(\mathrm{atm}\right)$	1–5 [21]
The doping level, DL	2–10 [22,26]
Thickness of the electrolyte, $t_{mem}\;\left(\mathrm{cm}\right)$	0.001–0.01 [21]

## Data Availability

Not applicable.

## References

[1] Reddy E.H., Monder D.S., Jayanti S. (2013). Parametric study of an external coolant system for a high temperature polymer electrolyte membrane fuel cell. Appl. Therm. Eng..

[2] Alpaydin G.U., Devrim Y., Colpan C.O. (2019). Performance of an HT-PEMFC having a catalyst with graphene and multiwalled carbon nanotube support. Int. J. Energy Res..

[3] Reddy E.H., Jayanti S. (2012). Thermal management strategies for a 1 kWe stack of a high temperature proton exchange membrane fuel cell. Appl. Therm. Eng..

[4] Devrim Y., Arica E.D. (2019). Multi-walled carbon nanotubes decorated by platinum catalyst for high temperature PEM fuel cell. Int. J. Hydrogen Energy.

[5] Oono Y., Sounai A., Hori M. (2009). Influence of the phosphoric acid-doping level in a polybenzimidazole membrane on the cell performance of high-temperature proton exchange membrane fuel cells. J. Power Sources.

[6] Lee D., Lim J.W., Lee D.G. (2017). Cathode/anode integrated composite bipolar plate for high-temperature PEMFC. Compos. Struct..

[7] Muthuraja P., Prakash S., Shanmugam V.M., Radhakrsihnan S., Manisankar P. (2018). Novel perovskite structured calcium titanate-PBI composite membranes for high-temperature PEM fuel cells: Synthesis and characterizations. Int. J. Hydrogen Energy.

[8] Li Q.F., Rudbeck H.C., Chromik A., Jensen J.O., Pan C., Steenberg T., Calverley M., Bjerrum N.J., Kerres J. (2010). Properties, degradation and high temperature fuel cell test of different types of PBI and PBI blend membranes. J. Membr. Sci..

[9] Selvakumar K., Prabhu M.R. (2018). Investigation on meta-polybenzimidazole blend with sulfonated PVdF-HFP proton conducting polymer electrolytes for HT-PEM fuel cell application. J. Mater. Sci.-Mater. Electron..

[10] Lobato J., Rodrigo M.A., Linares J.J., Scott K. (2006). Effect of the catalytic ink preparation method on the performance of high temperature polymer electrolyte membrane fuel cells. J. Power Sources.

[11] Durmayaz A., Sogut O.S., Sahin B., Yavuz H. (2004). Optimization of thermal systems based on finite-time thermodynamics and thermoeconomics. Prog. Energy Combust..

[12] Qin X.Y., Chen L.G., Ge Y.L., Sun F.R. (2013). Finite Time Thermodynamic Studies on Absorption Thermodynamic Cycles: A State-of-the-Art Review. Arab. J. Sci. Eng..

[13] Ge Y.L., Chen L.G., Sun F.R. (2016). Progress in Finite Time Thermodynamic Studies for Internal Combustion Engine Cycles. Entropy.

[14] Li C.J., Liu Y., Xu B., Ma Z.S. (2019). Finite Time Thermodynamic Optimization of an Irreversible Proton Exchange Membrane Fuel Cell for Vehicle Use. Processes.

[15] He S., Lin L.Y., Wu Z.X., Chen Z.M. (2020). Application of Finite Element Analysis in Properties Test of Finger-jointed Lumber. J. Bioresour. Bioprod..

[16] Yang J., Zhang Y.C., Zhou L., Zhang F.S., Jing Y., Huang M.Z., Liu H.B. (2021). Quality-related monitoring of papermaking wastewater treatment processes using dynamic multiblock partial least squares. J. Bioresour. Bioprod..

[17] Zhao X.Y., Huang Y.J., Fu H.Y., Wang Y.L., Wang Z. (2021). Deflection test and modal analysis of lightweight timber floors. J. Bioresour. Bioprod..

[18] Hu T.P., Yu Z., Guo L., Xu C.Y. (2019). Thermodynamic self-consistent dynamic model of wood dust explosion. J. For. Eng..

[19] Ji X.L., Yang P. (2020). The exploration of the slope displacement with vegetation protection under different rainfall intensity. J. For. Eng..

[20] Li X.S., Deng T.T., Wang M.H., Ju S., Li X.C., Li M. (2020). Linear positioning algorithm improvement of wood acoustic emission source based on wavelet and signal correlation analysis methods. J. For. Eng..

[21] Xu B., Li D.X., Ma Z.S., Zheng M., Li Y.J. (2021). Thermodynamic Optimization of a High Temperature Proton Exchange Membrane Fuel Cell for Fuel Cell Vehicle Applications. Mathematics.

[22] Guo Y.H., Guo X.R., Zhang H.C., Hou S.J. (2020). Energetic, exergetic and ecological analyses of a high-temperature proton exchange membrane fuel cell based on a phosphoric-acid-doped polybenzimidazole membrane. Sustain. Energy Technol. Assesments.

[23] Ye L., Jiao K., Du Q., Yin Y. (2015). Exergy Analysis of High-Temperature Proton Exchange Membrane Fuel Cell Systems. Int. J. Green Energy.

[24] Guo X.R., Zhang H.C., Yuan J.L., Wang J.T., Zhao J.P., Wang F., Miao H., Hou S.J. (2019). Energetic and exergetic analyses of a combined system consisting of a high-temperature polymer electrolyte membrane fuel cell and a thermoelectric generator with Thomson effect. Int. J. Hydrogen Energy.

[25] Liu G.K., Qin Y.Z., Yin Y.F., Bian X.Z., Kuang C.C. (2020). Thermodynamic modeling and exergy analysis of proton exchange membrane fuel cell power system. Int. J. Hydrogen Energy.

[26] Li D., Li S., Ma Z., Xu B., Lu Z., Li Y., Zheng M. (2021). Ecological Performance Optimization of a High Temperature Proton Exchange Membrane Fuel Cell. Mathematics.

[27] Huang Y.W., Ding H., Zou Y.F. (2020). Ecological Performance Analysis of an Integrated Proton Exchange Membrane Fuel Cell and Thermoelectric Devices. Int. J. Electrochem. Sci..

[28] Li C.J., Xu B., Ma Z.S. (2020). Ecological Performance of an Irreversible Proton Exchange Membrane Fuel Cell. Sci. Adv. Mater..

[29] Peng X.R., Zhang Z.K., Zhao L.Y. (2020). Analysis of Raman spectroscopy and XPS of plasma modified polypropylene decorative film. J. For. Eng..

[30] Yu P.J., Zhang W., Chen M.Z., Zhou X.Y. (2020). Plasma-treated thermoplastic resin film as adhesive for preparing environmentally-friendly plywood. J. For. Eng..

[31] Liu S.Q., Jia L.M. (2021). Review on sustainable development of forest-based biodiesel. J. Nanjing For. Univ. (Nat. Sci. Ed.).

[32] Xiong G.K., Li Y.Q., Xiong Y.Q., Duan A.G., Cao D.C., Sun J.J., Nie L.Y., Sheng W.T. (2021). Effects of low stand density afforestation on the growth, stem-form and timber assortment structure of Cunninghamia lanceolata plantations. J. Nanjing For. Univ. (Nat. Sci. Ed.).

[33] Yao J.J., Feng X.Q., Xiao H., Zheng Y., Zhang C.L. (2021). Improvement effects of different solid waste and their disposal by products on saline-alkali soil in Huanghua Port. J. Nanjing For. Univ. (Nat. Sci. Ed.).

[34] Zhang Z.G. (2020). Researches on green features and category architecture of green strategies of renewable-resource-based enterprises: A case study of forestry enterprise. J. Nanjing For. Univ. (Nat. Sci. Ed.).

[35] Zhou S.J., Wang P., Zhang M., Chen S.Z., Xu W., Zhu L.T., He X.Q., Gong S.R. (2021). Effects of atmospheric acid deposition on root physiological characteristics of Pinus massoniana seedlings. J. Nanjing For. Univ. (Nat. Sci. Ed.).

[36] Zhu P.H., Chen Y., Ji K.S. (2021). A review of terpene synthases and genes in Pinaceae. J. Nanjing For. Univ. (Nat. Sci. Ed.).

[37] Watowich S.J., Berry R.S. (1986). Optimal current paths for model electrochemical systems. J. Phys. Chem..

[38] Sieniutycz S. (2010). Thermodynamics of Power Production in Fuel Cells. Chem. Process. Eng.-Inz..

[39] Sieniutycz S., Poswiata A. (2012). Thermodynamic aspects of power production in thermal, chemical and electrochemical systems. Energy.

[40] Akkaya A.V., Sahin B., Erdem H.H. (2007). Exergetic performance coefficient analysis of a simple fuel cell system. Int. J. Hydrogen Energy.

[41] Guo X.R., Zhang H.C., Zhao J.P., Wang F., Wang J.T., Miao H., Yuan J.L. (2019). Performance evaluation of an integrated high-temperature proton exchange membrane fuel cell and absorption cycle system for power and heating/cooling cogeneration. Energy Convers. Manag..

[42] Dincer I., Hussain M.M., Al-Zaharnah I. (2005). Energy and exergy utilization in agricultural sector of Saudi Arabia. Energy Policy.

[43] Al-Sulaiman F.A., Dincer I., Hamdullahpur F. (2012). Energy and exergy analyses of a biomass trigeneration system using an organic Rankine cycle. Energy.

[44] Xu X.M., Zhang L., Jiang Y.P., Chen N. (2019). Active Control on Path Following and Lateral Stability for Truck-Trailer Combi-nations. Arab. J. Sci. Eng..

[45] Zhou W.L., Zheng Y.P., Pan Z.J., Lu Q. (2021). Review on the Battery Model and SOC Estimation Method. Processes.

[46] Chang C.C., Zheng Y.P., Sun W.M., Ma Z.S. (2019). LPV Estimation of SOC Based on Electricity Conversion and Hysteresis Characteristic. J. Energy Eng..

[47] Tian J., Zeng Q.K., Wang P., Wang X.Q. (2021). Active steering control based on preview theory for articulated heavy vehicles. PLoS ONE.

[48] Xu X.M., Lin P. (2021). Parameter identification of sound absorption model of porous materials based on modified particle swarm optimization algorithm. PLoS ONE.

[49] Xu X.M., Chen D., Zhang L., Chen N. (2019). Hopf Bifurcation Characteristics of the Vehicle with Rear Axle Compliance Steering. Shock. Vib..

[50] Ribeirinha P., Abdollahzadeh M., Pereira A., Relvas F., Boaventura M., Mendes A. (2018). High temperature PEM fuel cell integrated with a cellular membrane methanol steam reformer: Experimental and modelling. Appl. Energy.

